# Replicative Deformed Wing Virus Found in the Head of Adults from Symptomatic Commercial Bumblebee (*Bombus terrestris*) Colonies

**DOI:** 10.3390/vetsci8070117

**Published:** 2021-06-23

**Authors:** Giovanni Cilia, Laura Zavatta, Rosa Ranalli, Antonio Nanetti, Laura Bortolotti

**Affiliations:** CREA Research Centre for Agriculture and Environment, Via di Saliceto 80, 40128 Bologna, Italy; giovanni.cilia@crea.gov.it (G.C.); laura.zavatta@crea.gov.it (L.Z.); rosa.ranalli@crea.gov.it (R.R.); laura.bortolotti@crea.gov.it (L.B.)

**Keywords:** bumblebee, commercial *Bombus terrestris*, spillover, DWV, honey bee pathogens, replicative virus, strand-specific RT-PCR

## Abstract

The deformed wing virus (DWV) is one of the most common honey bee pathogens. The virus may also be detected in other insect species, including *Bombus terrestris* adults from wild and managed colonies. In this study, individuals of all stages, castes, and sexes were sampled from three commercial colonies exhibiting the presence of deformed workers and analysed for the presence of DWV. Adults (deformed individuals, gynes, workers, males) had their head exscinded from the rest of the body and the two parts were analysed separately by RT-PCR. Juvenile stages (pupae, larvae, and eggs) were analysed undissected. All individuals tested positive for replicative DWV, but deformed adults showed a higher number of copies compared to asymptomatic individuals. Moreover, they showed viral infection in their heads. Sequence analysis indicated that the obtained DWV amplicons belonged to a strain isolated in the United Kingdom. Further studies are needed to characterize the specific DWV target organs in the bumblebees. The result of this study indicates the evidence of DWV infection in *B. terrestris* specimens that could cause wing deformities, suggesting a relationship between the deformities and the virus localization in the head. Further studies are needed to define if a specific organ could be a target in symptomatic bumblebees.

## 1. Introduction

The deformed wing virus (DWV) belongs to the Picornaviridae family, within the Iflavirus genus, with a positive-sense ssRNA [1,2]. As an *Apis mellifera* pathogen, DWV is deemed to be involved in colony losses and, as such, a significant impact to the ecosystem [3,4]. The virus is spread globally [1,2,5,6], and DWV infections are generally detected due to the presence of symptomatic honey bees, characterized by deformed or missing wings and shortened abdomens [1]. Presently, three genetic DWV variants are acknowledged and named as type A, B, and C [7,8], with type A being the most prevalent [8].

In honey bees, DWV is usually associated with *Varroa destructor* infestations, even if, in some cases, the virus was detected in *Varroa*-free bees [9]. Within the colony, it is transmitted by the punctures produced in both the juvenile and adult stages [10], but the infection may also spread horizontally by bee-to-bee contact [11,12,13,14,15] and ingestion of contaminated food [16,17,18]. However, due to the multiple possible transmission routes, DWV may spillover to other sympatric Hymenopterans [19,20,21], including species used in commercial pollination [22,23,24,25,26] and beetles [27].

Bumblebees are among the most common and widespread bee pollinators, mainly in temperate and cold areas. Due to their unique ability to pollinate tomato crops, they began to be bred and sold in many countries for pollination purposes [28]. Bumblebee rearing and trading date back to the 1980s, when the first company for the commercial rearing of *B. terrestris* was founded in Belgium. Bumblebees were initially used for tomato pollination in greenhouses, but later they extended to other crops both in open fields and greenhouses. Presently, bumblebees are commercially bred in all continents, except Africa, with a global sales volume exceeding 1,000,000 colonies per year. They are sold worldwide, with the main exception of mainland Australia. Due to the emerging risks in the marketing of non-native species and the resulting restrictions, many local species of bumblebee began to be bred for pollination in the countries of origin, but *B. terrestris* always remains the most used and widespread in Europe, North Africa, and West Asia [29,30].

One of the main concerns in the commercialization of bumblebees is the spread of diseases and pathogens [31]. Commercial bumblebee colonies can suffer from various diseases and pests, which can affect their survival. Those that can cause major problems are the protozoan *Nosema bombi*, the tracheal mite *Locustacarus (Bombacarus) buchneri,* the Trypanosomatidae *Crithidia bombi*, and the brood parasites *Melittobia acasta* and *M. chalybii*, whose massive presence in breeding facilities requires the stamping out of the infected colony and, in the most severe infestations, of the entire production stock [32,33,34,35]. Other less dangerous but still problematic pests are the pyralid moths *Vitula edmandsii* and *Plodia interpunctella* whose larvae feed primarily on bumblebee food stores but occasionally also on bumblebee brood [29].

Thus far, viral infections have not been considered major threats for wild and commercial bumblebees. However, recent findings show the presence of replicative honey bee viruses in wild bees, including bumblebees. The infection with some of these viruses proved to cause negative consequences in *B. terrestris*, such as reduced fecundity and colony founding associated with Kashmir bee virus (KBV), reduced fecundity due to Israeli acute paralysis virus (IAPV), and mortality due to acute bee paralysis virus (ABPV) [36,37,38,39]. Conversely, *B. terrestris* infection with DWV has been found to result in wing deformities and mortality in a limited number of individuals [40,41]. A recent investigation showed that DWV could replicate in *Bombus* pupae after artificial infection and larval feeding of virus-contaminated food, but none of the infected bumblebees showed signs of wing deformities [23].

Since bumblebees are not affected by Varroa infestations, it remains unclear how the infection occurs under natural conditions, although DWV-positive *Bombus* sp. Were found in areas with a high DWV prevalence in Apoidea species [40,42,43,44]. Possibly, the virus transmission between sympatric pollinator species is mediated by shared floral resources such as pollen [16,24]. Similarly, in commercial breeding, the most likely infection route is by feeding the bumblebees with pollen from honey bee colonies. In effect, batches of honey bee pollen used to feed bumblebees revealed the presence of several pathogens (*Crithidia* spp., *Ascosphaera bombi*, *A. apis, Nosema ceranae, Nosema thomsoni, Microsporidium* sp. Oise), including viruses (sacbrood virus—SBV, DWV, IAPV, and chronic bee paralysis virus—CBPV) [45]. This study aimed to investigate the DWV distribution in the body of symptomatic and asymptomatic *Bombus terrestris* specimens reared in a commercial colony after the first detection in a worker with relevant wing deformities.

## 2. Materials and Methods

### 2.1. Sample Collection

**Figure 1:**
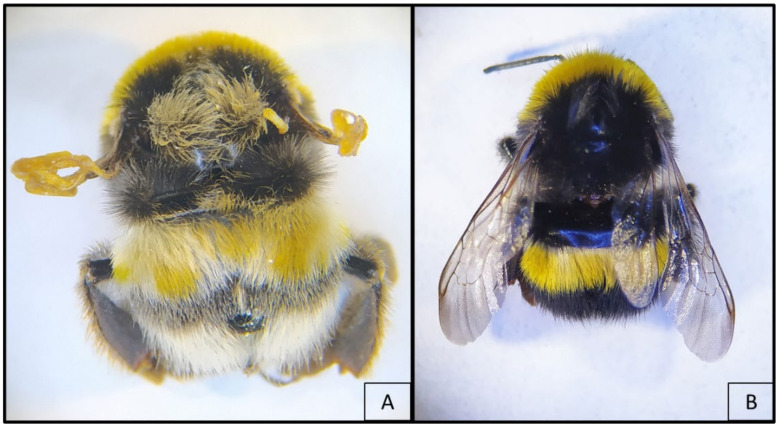
*Bombus terrestris* workers with crippled (**A**) and normal (**B**) wings. Individual A shows yellow and white patches of anomalous pigmentation.

On 15 January 2021, three commercial bumblebee colonies (*B. terrestris*) arrived from a North European breeding company to our laboratory (Bologna, Italy) for an experimental test requiring colonies in an advanced stage of development. The colonies contained about a hundred workers and pupal stages of gynes and males. Moreover, they appeared healthy and in good condition, although the founder queen was found dead in all of them. The colonies were kept in a climate room for a total of three weeks, under constant environmental conditions of temperature (25 ± 1 °C) and relative humidity (40 ± 10%). Darkness (0:24 L:B) was maintained throughout the rearing period, and all the laboratory manipulations were conducted with the aid of red lights [46]. Flight activity was not allowed. The colonies had arrived from the breeding company with food supplies that were integrated onsite with frozen pollen from local honey bee colonies. On 17 January 2021, a routine check allowed for the detection in one of the colonies of one worker with crippled wings (Figure 1).

As this observation was compatible with a DWV infection, a sampling plan created to collect and analyse all kinds of individuals present in the colonies. Accordingly, the following asymptomatic individuals were sampled from each colony: five gynes, five workers, five males, five pupae, five larvae, and three pools of eggs (*n* = 5). All the adults that were found with crippled wings were also individually collected.

### 2.2. Extraction of Total RNA

Before dissection and extraction of viral RNA, all samples were washed, through full immersion in 95% ethanol for 10 s to remove any external viral contamination that may have been present. Each adult was dissected under sterile conditions with a scalpel to exscind the head from the rest of the body (thorax and abdomen), which were collected in separate 2-ml microtubes. To avoid possible cross contamination, new scalpels were used for each individual. The juvenile stages (pupae, larvae, and the pool of eggs) were introduced undissected into separate 2-ml microtubes. Beads and 80 μL of Lysis Buffer provided by GeneJET RNA Purification Kit (ThermoFisher Scientific, Waltham, MA, USA) were added to each sample, which was crushed with a TissueLyser II (Qiagen, Milan, Italy) for 3 min at 25 Hz, as previously described [47,48].

Total RNA was extracted from each sample with GeneJET RNA Purification Kit (ThermoFisher Scientific, Waltham, MA, USA) following the manufacturer’s instruction. All samples were eluted in 100l RNase-free water. The RNA extracts were stored at −80 °C until use. High pure sterile DNA- and RNA-free water was used as a negative control in all analytical steps.

### 2.3. qRT-PCR Assays to Detect and Quantify the Deforming Wing Virus (DWV)

The extracted RNAs were analysed by qRT-PCR to detect and quantify the presence of DWV in bumblebees. Primers amplified a 132-bp fragment within the highly conserved region coding for the RNA-dependent RNA polymerase (*Rd-Rp)* commonly expressed in all virus variants. The sequences were: DWV Fw 5′- TTTGACATTGAGCTACAAGACTCG-3′ (nt. 8685–8708), DWV Rev 5′- ACAATCCGTGAATATAGTGTGAGG-3′ (nt. 8816–8793) [16]. The viral genomes were amplified using Power SYBR^®^ Green RNA-to-Ct™ 1-Step Kit (ThermoFisher Scientific, Waltham, MA, USA), following the manufacturer’s instruction. The qPCR assay was performed on Applied Biosystems^®^ 7500 fast and 7500 Real-Time PCR (ThermoFisher Scientific, Waltham, MA, USA). For the target gene, a total reaction volume of 20 μL was used following the protocols previously described [16,19].

The successful amplification of reference gene β-Actin, with the previously described primers [49], was used to confirm the sample integrity from the RNA extraction to the qPCR analysis and to normalize the results to reach an absolute quantification.

Virus loads were quantified with absolute quantification of number of DWV copies in each ng of RNA (copies/ng RNA). The amplified fragment was gel purified using GeneJET Gel Extraction and DNA Cleanup Micro Kit (ThermoFischer Scientific, Waltham, MA, USA) and cloned using CloneJET PCR Cloning Kit with DH10B Competent Cells (ThermoFischer Scientific), following the manufacturer’s instructions, and sequenced (BMR Genomics, Padova, Italy). Following plasmid DNA removal by RNase-free DNase treatment (RNase Free DNase Set, Qiagen, Hilden, Germany), the transcript was purified and concentrated with the RNeasy Minelute Cleanup Kit (Qiagen, Hilden, Germany) and quantified with the Quant-iT™ RiboGreen™ RNA Assay Kit (ThermoFisher, Waltham, MA, USA). A standard curve was created with six tenfold dilutions of cloned RNA fragment (2 × 10^5^ to 2 copies/ng) [16].

### 2.4. Strand-Specific RT-PCR

The DWV replication was evaluated through a strand specific RT-PCR using specific primers Fw 8450: 5′- TGGCATGCCTTGTTCACCGT-3′ (nt. 8450–8469) or Rev 8953: 5′-CGTGCAGCTCGATAGGATGCCA-3′ (nt. 8953–8932), which amplify a 504-bp fragment of the *Rd-Rp*, as previously described [16]. All amplicons were visualized on a 1.5% agarose gel.

The DWV sequence described in this study was submitted to the GenBank database under the accession number MZ222242.

### 2.5. Phylogenetic Analysis

The strand specific RT-PCR-obtained amplicons were sequenced (BMR Genomics, Padua, Italy) and analysed using BLASTn to standard databases with default parameters for megablast [50]. The sequences with a high Max Score and a Query ≥70% in the BLAST analysis were selected to build the phylogenetic tree. The phylogenetic analysis was performed by the maximum likelihood method based on the Tamura–Nei model with a bootstrap test using MEGA software [51].

### 2.6. Statistical Analysis

As the batch of colonies was provided by the same producer and all of them were prevented from flying, the ‘colony’ as an explanatory factor for the DWV titre was considered unimportant. This consideration elicited the decision to pool the data together by the kind of individual, as if they all belonged to the same colony.

The results were analysed with a parametric approach. Before the statistical analysis, significant violations to the assumptions of parametric tests were removed by log transformation of data. However, text and illustrations report untransformed data.

The number of DWV copies detected in the dissected body parts from the same adult was summed up to calculate the total individual DWV titre. This was considered as the dependent variable in a one-way analysis of variance (ONE-WAY ANOVA) with the ‘kind of individual’ (deformed adults, gynes, workers, males, pupae, larvae, and eggs) as the categorical factor.

A factorial ANOVA was conducted on the amount of DWV copies detected in the adults against the categorical independent variables ‘kind of individual’ (deformed adults, gynes, workers, males) and ‘body part’ (head, rest of the body). The interaction between the two factors mentioned above was also calculated.

When the F-test resulted in a significant effect, a pairwise Newman-Keuls post hoc test was conducted to spot significant differences between the groups.

For all tests, the protection level from statistical Type I error was set at *p* ≤ α = 0.05.

## 3. Results

**Figure 2:**
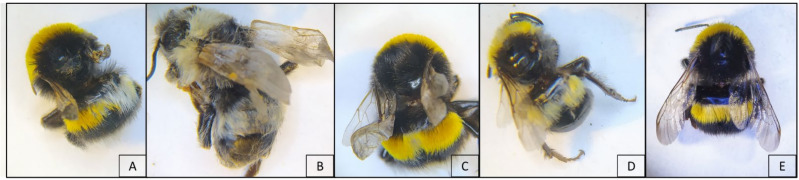
Evidence of several degrees of wing deformities in symptomatic DWV infected *Bombus terrestris*. Wings of adult bumblebees from the inspected colonies: bilateral (**A**,**C**) and unilateral (**D**) deformity in workers; bilateral deformity in newly hatched male (**B**); asymptomatic worker (**E**). Deformed individuals show yellow and white patches of anomalous pigmentation.

Wing deformity (Figure 2) was detected in adults of all colonies. In detail, the deformities were found, respectively, in one and six workers of two colonies and in four workers and one newly hatched male (Figure 2B) of the third one. 

**Figure 3:**
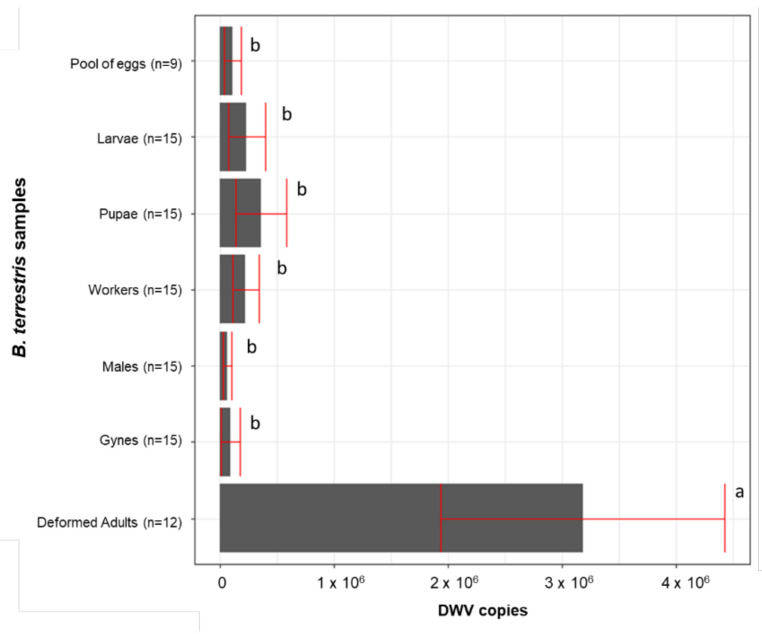
DWV titre of deformed and asymptomatic adults and juvenile individuals. Averages +/− standard error are shown. The same letter indicates a nonsignificant difference.

All samples scored positive for DWV, with a number of genomic copies ranging from 2.01 × 10^2^ to 5.10 × 10^6^. The viral titre varied significantly in the different kinds of individuals that were sampled (F(6, 69) = 6.130, *p* = 0.000); a Newman-Keuls test showed that the amount of DWV copies was significantly higher in the deformed adults compared to the asymptomatic adults and the eggs, larvae, and pupae). Among the nondeformed individuals, no significant differences were detected despite belonging to different stages, sexes, and castes (Figure 3).

**Figure 4:**
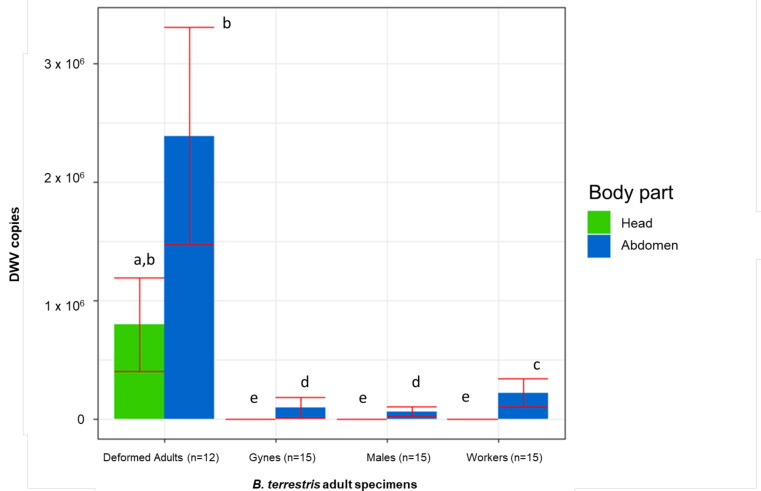
DWV copies detected in both head and rest of the body of deformed and asymptomatic *B. terrestris* adults. Averages (columns) and standard errors (vertical bars) are shown. The same letters highlight nonsignificant differences (*p* ≤ 0.05).

In the adults, the DWV titre was significantly influenced by the kind of individual (F(3, 66) = 127.687, *p* = 0.000), the body part (F(1, 66) = 337.772, *p* = 0.000), and their interaction (F(3, 66) = 36.342, *p* = 0.000). A Newman-Keuls test did not show a significantly different DWV abundance in the head and the rest of the body of deformed individuals. However, all the considered types of asymptomatic adults had significantly fewer copies in their heads. Furthermore, in both the heads and the rest of the body of deformed individuals, the number of DWV copies was significantly higher compared to the asymptomatic adults (Figure 4).

**Figure 5:**
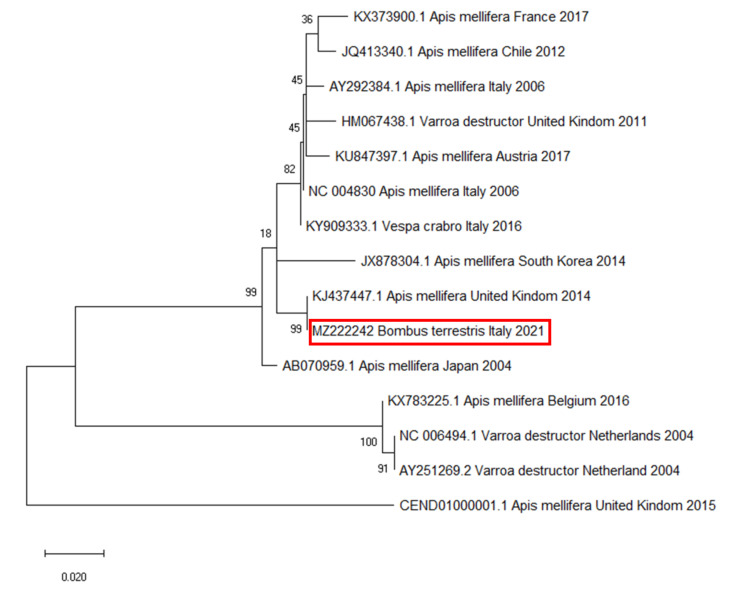
Molecular phylogenetic analysis for RNA-dependent RNA polymerase of deformed wing virus (DWV) by maximum likelihood method. The evolutionary history was inferred using the maximum likelihood method based on the Tamura–Nei model. The branch lengths of the tree measured the number of substitutions per site. The analysis involved 28 nucleotide sequences. There were 255 positions in the final dataset. Accession number, host, state, and year of available GenBank DWV sequences are shown. DWV sequence accession numbers are reported and associated with year and site of origin. The DWV sequence obtained from the tested *B. terrestris* samples is highlighted by a red box.

The strand-specific PCR demonstrated the active DWV replication in all tested samples. The BLAST analysis performed on the obtained amplicons confirmed the specificity of the sequences, with high similarity (99% of percent identity, 0.0 of E-value, 100% of Query Cover) to specific DWV genomes deposited in GenBank. The same sequence was recorded in all positive samples. The phylogenetic analysis and pairwise distance analysis indicated the highest homology to DWV strains isolated from *A. mellifera* in the United Kingdom (Figure 5).

## 4. Discussion

Several studies highlight that bees other than *A. mellifera* can be infected by the DWV [22,24,25,26,41,52]. Additionally, the virus has been detected in the hornets *Vespa crabro* and *Vespa velutina* [19,20], wasp *Vespula vulgaris* [53], invasive ant *Linepithema humile* [21], and beetle *Aethina tumida* [27,54,55]. This shows that spillover may occur between both close superfamilies (Apoidea, Vespidae) and insect species belonging to relatively distant groups. 

In *Bombus* spp., DWV has been found in several instances worldwide and considered a significant factor of decline in both wild and managed bumblebee populations [56,57]. DWV infections have been reported in *B. terrestris* [25,40,52,58,59,60], *B. pascuorum* [25,41,52,58,60], *B. impatiens* [24,25,61,62,63], *B. atratus* [64,65], *B. vagans* [24,62], *B. huntii* [63], *B. ruderatus* [59], *B. ternarius* [24], *B. lapidarius, B. lucorum*, and *B. monticola* [40].

Despite the fact that DWV infections are a frequent occurrence in *B. terrestris* [22,25], symptomatic adults with crippled and deformed wings have been seldom reported. To the best of our knowledge, only *B. terrestris* queens and *B. pascuorum* workers have been found showing symptoms linked to DWV infection, reporting replicative viral RNA in the thorax and abdomen [41]. Moreover, in asymptomatic *B. hunti*, DWV was found in the brain and antennae of wild males and reared males and workers, even if it was not found to be replicative [63].

In *A. mellifera*, DWV is chiefly transmitted by the Varroa mites [1,6,15,17,22]. Missing a known vector, in bumblebees, the infections are more likely to propagate by a feeding route through the consumption of contaminated food and trophallaxis.

Indeed, commercial bumblebee producers may use honey bee collected pollen as a protein source to feed the queens and support the colony development [24,66,67]. In this respect, fresh frozen pollen has a shown higher effectiveness compared to dry pollen, which makes the first generally preferred to feed both queens and developing colonies [68]. However, the pollen collected by the honey bees may contain pathogens, including viruses [45], which may have likely played a role in developing the infections detected in this study. Although the investigated colonies were fed fresh frozen pollen in our laboratory, they already had adults with crippled wings when they arrived, indicating that possible pollen-mediated DWV infections must have occurred at the breeding site. Furthermore, flowers may become contaminated with DWV when visited by infected honey bees, thus representing sites for interspecific transmission to other pollinators, wild bumblebees included [16,17,52,69].

Additionally, newly emerged honey bee workers may be used in commercial breeding to stimulate bumblebee queens to initiate their nesting activity after hibernation [41], but this method is rarely used as it is less effective in inducing oviposition than the use of the bumblebee pupa [70]. Finally, the vertical transmission is also not to be excluded because a DWV-positive queen could deposit infected eggs, from which infected adults can be born, as suggested by the deformed and positive newly emerged male collected.

The total viral load found in each investigated *B. terrestris* individual is comparable to values measured in asymptomatic honey bees (from 10^2^ to 10^6^ copies) and lower than the viral titre of symptomatic honey bees (> 10^7^ copies) [16]. In all investigated samples, DWV was found in replicative form, demonstrating its adaptability and capability to replicate in *Bombus* cells [22,41]. Nonsignificant differences in DWV titre were observed in adults considering the head and the rest of the body separately. As for DWV-positive honey bees [41], the symptomatic bumblebees investigated here were characterized by virus presence in the head. Additionally, our results highlighted a relationship between deformities in adults and the presence of DWV in the head. Previously, the *B. terrestris* and *B. pascuorum* specimens with crippled wings only resulted positive for the presence of DWV in the thorax and abdomen, but their head scored negative [41]. Additionally, in asymptomatic artificially infected *B. huntii* specimens, the DWV was found in the antennae and brain [63]. This study could not clarify whether a relationship existed between DWV infection and abnormal pigmentations in symptomatic individuals.

Although other causes of wing malformation cannot be excluded, they should be seen as most unlikely. Upon arrival at the laboratory, the three colonies showed healthy, well-developed, and without signs of mishandling or malnutrition. The fact that in all the colonies the founder queen had died is not uncommon at this stage of colony development, although conventionally it makes the colonies unsuitable for commercialization. In many insect species, the development of deformed adults can also be caused by thermal shock, but in bumblebee colonies, this usually occurs at the early stages of development, when there are still only few workers tending the brood [71]. However, during the three weeks of observation by our laboratory, the colonies were kept at a constant temperature and humidity and carefully manipulated.

Finally, the phylogenetic analysis highlighted that the obtained DWV amplicons showed high similarity (99% identity) with a DWV sequence isolated from the honey bees in the United Kingdom in 2014 [9], suggesting that DWV could be associated with the commercial origin of colonies.

## 5. Conclusions

The results of this study, other than indicating evidence of DWV infection in *B. terrestris* specimens causing wing deformities similar to the clinical lesion in honey bees, suggest a relationship between the wing deformities and the virus localization in the head. Further studies are needed to define if a specific organ could be a target in symptomatic bumblebees. Furthermore, the high similarity of sequenced amplicons with DWV isolated from honey bees in the United Kingdom indicates the North European origin of the virus and suggests that transmission could occur by contaminated pollen administered to bumblebees. The finding of adults with deformed wings and the premature death of the queen raises the question of to what extent the presence of the virus can be associated with a reduction of vitality at the individual level.

Several studies demonstrate that commercially produced bumblebee colonies can carry multiple infectious parasites, posing a significant risk to other native and managed pollinators through pathogen spillover [72]. Currently, commercial colonies must be accompanied by a parasite-free certification, and rearing facilities may be subject to inspection by the national veterinary services. If the colonies are intended for exportation, the certificates may also cover honey bee parasites and pests (e.g., *Varroa destructor, Tropilaelaps* spp., *Aethina tumida*, and American foulbrood) but not honey bee viruses.

The geographical origin of the DWV strains found in our colonies and the fact that most commercial *B. terrestris* breeding is located in Northern Europe raise concerns about the possible future increase of virus spread by long-range export of infected colonies. This advocates for intensified controls in bumblebee rearing operations and the application of preventive measures against virus spread, such as pollen sterilization by gamma radiation, which has already been proven effective against IAPV present in honey bee-collected pollen [73].
